# Titanium-based hybrid coatings grown using ALD/MLD onto AZ31 screw-like supports for implantable systems†

**DOI:** 10.1039/d4ra07952c

**Published:** 2025-04-07

**Authors:** R. M. Silva, F. J. Oliveira, M. F. Lima, N. A. Silva, G. Miranda

**Affiliations:** a CICECO-Aveiro Institute of Materials, Department of Materials and Ceramic Engineering, University of Aveiro 3810-193 Aveiro Portugal rmsilva@ua.pt gmiranda@ua.pt; b Life and Health Sciences Research Institute (ICVS), School of Medicine, University of Minho Campus de Gualtar 4710-057 Braga Portugal; c ICVS/3B's-PT Government Associate Laboratory Braga Guimarães Portugal

## Abstract

Atomic/molecular layer deposition (ALD/MLD) is ideally suited for addressing the challenges faced by the new generation biomedical technologies through surface and interface modification with organic–inorganic hybrid coatings, which are emerging as an alternative to inorganic coatings. In this study, we present a feasible strategy for modifying the surface of magnesium alloy (AZ31) screw-like substrates with titanium-based hybrid coatings, using titanium tetraisopropoxide as the metal-bearing precursor, and a simple aliphatic bi-functional alcohol, such as ethylene glycol, as the organic precursor. Results demonstrated that the titanium-based hybrid coating was evenly distributed without obvious defects on the AZ31 screw-like substrates while providing physical protection. In addition, the cytocompatibility of the titanium-based hybrid coating was validated through the cytotoxicity assay, revealing its potential for future biomedical applications.

## Abstract 2

Atomic/molecular layer deposition (ALD/MLD) is ideally suited for addressing the challenges faced by the new generation biomedical technologies through surface and interface modification with organic–inorganic hybrid coatings.

## Graphical abstract

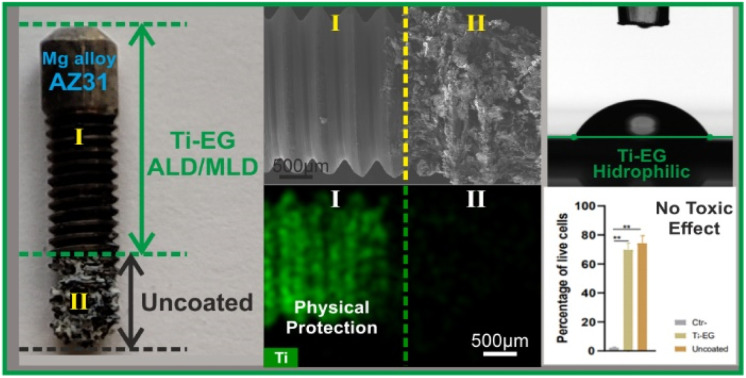

## 1. Introduction

Atomic layer deposition (ALD) is a promising technique for modifying or controlling the activity of solid surfaces through the deposition of homogeneous and conformal inorganic coatings (binary metal oxides) due to its self-limiting surface reactions,^1^ yielding a desired functionality to the surface that often requires engineering of the material structures in nanoscale. The surface-engineered property plays a very important role in many applications for biomaterials, ranging from medical devices to pharmaceuticals.^2–6^ Interestingly, ALD is gradually integrating into the medical and pharmaceutical fields since the main driving force of ALD is the fabrication of microelectronic and nanostructured materials.^7,8^ In parallel to the ALD of inorganic coatings, molecular layer deposition (MLD) was introduced as an organic counterpart of ALD, where functional organic monomers are used to grow purely organic coatings. However, there is a new trend to bridge the ALD with MLD for advanced hybrid organic–inorganic materials, with new and/or altered properties when compared to the individual or parent components, for a variety of emerging applications.^9–12^ Various organic–inorganic hybrid thin films, for instance, the so-called “metalcones”, have been prepared using ALD/MLD. This is a well-established process based on the reaction between organometallic precursors and aliphatic bi-functional alcohols (*e.g.*, ethylene glycol).^9^ The term “metalcones” can be distinguished based on the chosen metal, with reference to the silicones, which show a similar molecular composition.^11^

Herein, we report the synthesis of “titanicones” hybrid organic–inorganic thin films formed by the reaction of titanium(iv) tetraisopropoxide (TTIP) and ethylene glycol (EG) to interact with biological systems. The titanium-based hybrid thin films can be considered a promising candidate to replace traditional titanium oxide (TiO_2_) thin films in biomedical uses due to their similarity with TiO_2_.^13^ TiO_2_ thin films present excellent biocompatibility and chemical stability, and these nanomaterials have been used as coatings for metallic bioimplants and porous materials.^14–17^ Webster *et al.*,^18^ in 1999, found that nanoscale ceramic materials (Al_2_O_3_; TiO_2_) were more conducive to the adhesion and growth of osteoblasts than micron-scale surface structures, triggering the subsequent studies on nanoscale surface modifications. TTIP was chosen as the metal source due to its chemical stability, safety and low-temperature deposition range.^19^ In addition, the TTIP does not produce corrosive by-products during the deposition process, which is very desirable for the practical application pursued in the present work. It has been used to grow both inorganic TiO_2_ thin films as well as hybrid organic–inorganic thin films by replacing the water with organic molecules having two functional groups (*e.g.*, aliphatic organic diols and/or aromatic dicarboxylic acids), and they need to be reactive toward the inorganic metal precursor at suitable deposition temperatures.^13,20^ To the best of our knowledge, no reports are available on the ALD/MLD coatings of magnesium (Mg) alloy substrates, such as AZ31 (A, aluminum and Z, zinc) alloying elements with inorganic–organic hybrid thin films. The Mg-based alloys are interesting supports for such hybrids as they present excellent biocompatibility and biodegradability over their non-metallic counterpart materials. Therefore, the AZ31 alloy has been widely used in orthopedic applications due to its suitable properties, *e.g.*, high mechanical strength and lightweight.^21–25^ In other words, the microstructure of the alloy determines its load-bearing properties, while the biological properties are governed by its surface chemistry and degradation behavior, as the biological environment is considered to be extremely aggressive. Obviously, its relatively poor degradation resistance is one of the existing challenges that still require intensive research for improvement. Recently, coatings have been proven to provide an effective barrier between the substrate and the simulated biological environments. In this context, the ALD of TiO_2_, ZrO_2_, and HfO_2_ layers deposited on the AZ31 alloy has been explored as a protection barrier and assessed by *in vitro* electrochemical investigations.^26–31^ Such inorganic coatings are in the range of 100 nm, either as an individual layer or as multilayers and in an amorphous state. It is anticipated that this study will fill the knowledge gap regarding the key factors that impact the cytocompatibility response, which will facilitate the further application of these hybrid thin films in biomaterial applications.

## 2. Experimental details

### 2.1. Materials and chemicals

#### Substrates

Single-side polished silicon (SSP) Si (100) with thermally grown SiO_2_ wafers (300 nm) were purchased from SILTRONIX and used after cleaning in a ‘piranha’ solution. The sample substrates were cleaved from intact Si wafers into 1.5 cm × 1.5 cm squares (Fig. S1†).

A commercial extruded magnesium alloy rod (AZ31-Mg 96/Al 3/Zn 1, diameter 3 mm, Goodfellow) was employed for the fabrication of screw-like substrates (Fig. S2†). The AZ31 rod was cut into 1.3 cm of length and screw thread (thread cutter, M3 × 0.5 mm, pitch). The AZ31 screw-like substrates were further sonicated in isopropanol for 15 min at RT for proper substrate cleaning to free the surface of residues and impurities from the threading process. Subsequently, the AZ31 screw-like substrates were blown dry with compressed air.

#### Chemicals

Titanium(iv) tetraisopropoxide (TTIP, STREM Chemicals, 98% purity), ethylene glycol (EG, Alfa Aesar, 99%), phosphate buffered saline tablet (PBS, Sigma-Aldrich), ultrapure water (obtained from a Milli-Q purification system), Ar gas (Ar, Air Liquide, 99.9999% purity), and isopropanol (Sigma-Aldrich, 100%). All chemicals were used as received.

### 2.2. Ti–EG ALD/MLD

**Fig. 1:**
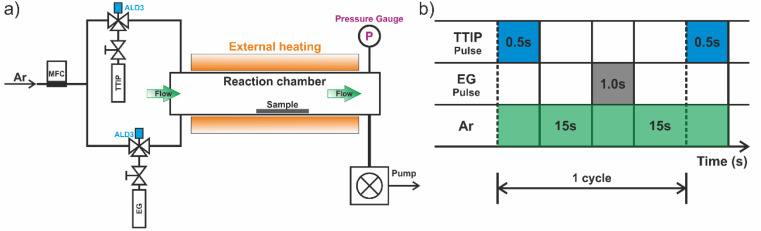
(a) Schematic of the thermal-ALD tubular reactor and (b) representative time sequence of the precursor dosing and Ar flows during the ALD/MLD process. The deposition zone of the reactor was defined by the heated zone of the tube furnace.

The hybrid thin film depositions were performed by combining the ALD and MLD cycles on a tubular custom-made cross-flow ALD reactor (Fig. 1a), controlled by a DAYSLab software, working in a continuous mode at 105 °C using TTIP and EG as precursors. The TTIP precursor was placed in a stainless steel canister filled with Ar in an Ar glove box and sealed with a manual valve. The canister was kept under vacuum once attached to the ALD chamber and maintained at 80 °C. The deionized water canister was held at room temperature (RT). Pure Ar, supplied *via* a mass flow controller, was used as a carrier gas at a constant flow of 50 sccm, leading to an operating pressure of 1.8 torr during the precursor pulse timing. The precursors were introduced near the front door of the tubular reactor by two individual inlets to avoid cross-contamination. The reactor was pumped using a mechanical pump (Pfeiffer Vacuum) with a molecular sieve trap (MDC Vacuum Products) to trap any organic effluent and protect the pump during deposition, and the pressure was monitored using a Pirani gauge. Prior to deposition and during heating of the reaction chamber up to the synthesis temperature, the substrates were purged with pure Ar (50 sccm of Ar) gas; this stage has a duration time of 60 min, allowing the stabilization of the reaction chamber temperature. Precursor pulse times of 0.5 s and 1.0 s for TTIP and EG were used, respectively. The reactant pulses were separated by 15 s (Fig. 1b). TTIP was held at 80 °C, but EG was maintained at 90 °C due to its low volatility. All steps were automatically controlled *via* fast ALD pneumatic valves (Swagelok ALD3). The delivery lines of the circuit were wrapped in a heating tape covered by thermal insulation tape and heated to 100 °C to prevent the precursors from condensation on the internal walls of the delivery lines throughout the deposition process.

**Fig. 2:**
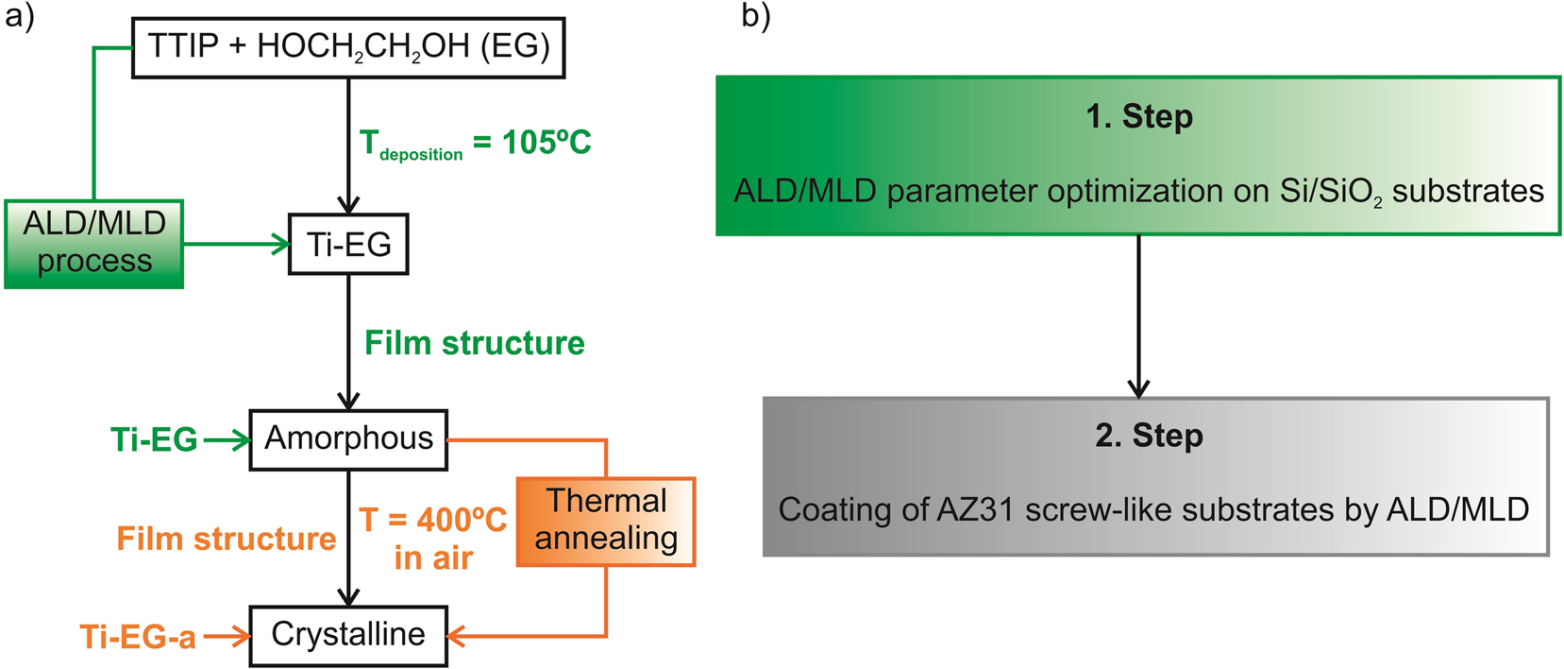
(a) Schematic of the synthetic procedure for Ti-EG deposition as well as the *ex situ* thermal annealing step. (b) Schematic illustration of the workflow representing the obtained results: (1) Si/SiO_2_ and (2) AZ31 screw-like substrates.

Several samples were prepared. Fig. 2a shows a schematic illustration of the Ti–EG depositions on the Si/SiO_2_ substrates, including the *ex situ* thermal annealing step. Before coating the AZ31 screw-like substrates, Si/SiO_2_ substrates were used to optimize the ALD/MLD parameters and the characterizations were performed (Fig. 2b).

*Ex situ* thermal annealing in the air: after the ALD/MLD process, the samples (Si/SiO_2_/Ti–EG and AZ31/Ti–EG) were loaded into a quartz boat and annealed in an air atmosphere at 400 °C using a high-temperature tubular furnace. During annealing, the temperature was ramped up at a rate of 1 °C min^−1^ to 400 °C. This temperature was held for 4 hours before the heating source was turned off, and then the samples were cooled to room temperature. The annealed samples are labeled as Ti–EG-a.

### 2.3. Characterization

The crystallinity of the thin films was assessed using the grazing incidence X-ray diffraction (GIXRD) mode on a Philips X'Pert MRD diffractometer with Cu K_α_ (*λ* = 1.5418 Å) radiation and a point detector. The X-rays were generated by applying a 45 kV voltage on the Cu anode at a current of 40 mA. Each sample was carefully aligned such that the beam was centered on the sample at a consistent height relative to the incident X-ray beam. The measurement range of the angle 2*θ* was from 3° to 80°, with a step size of 0.02°. The thickness of the Ti–EG and Ti–EG-a films deposited on the Si/SiO_2_ wafer substrates was measured by X-ray reflectivity (XRR) on the same diffractometer. To enhance the intensity of the X-rays diffracted from the thin films and avoid signals from the Si/SiO_2_ substrate, the GIXRD measurements were conducted at a low grazing angle of 0.5°. The powder XRD pattern of the degradation products was recorded on a Panalytical Empyrean equipped with a Cu K_α_ (*λ* = 1.5418 Å) X-ray tube (40 mA, 45 kV). The range of measurements of the angle 2*θ* was from 5° to 100°, with a step size of 0.03°.

The Raman spectra were recorded at room temperature using a Raman spectrometer (Jobin Yvon T64000) with an excitation wavelength of 532 nm and calibrated with the phonon mode of Si at 521 cm^−1^, with a focused laser spot size of ∼1 μm. In particular, the modification of the AZ31 screw-like surface after the hybrid thin film deposition was also analyzed using Raman spectroscopy.

The chemical bonding of the films was investigated using attenuated total reflectance Fourier transform infrared (ATR-FTIR, Bruker Tensor 27 at 4 cm^−1^ resolution) spectroscopy. The interference from the Si substrate was suppressed by subtracting the ATR-FTIR spectrum of the bare Si/SiO_2_ substrate (reference sample) from the ATR-FTIR spectra of the samples.

The morphology of the samples was characterized using scanning electron microscopy (SEM) on a Hitachi SU-70 microscope operated in the secondary electron mode at 15 kV and equipped with energy-dispersive X-ray spectroscopy (EDX). The samples were sputter-coated with a thin layer of carbon.

The surface topography of the films was analyzed using atomic force microscopy (AFM) with a Ntegra Prima setup (NT-MDT) in the tapping mode under ambient conditions. A silicon cantilever (Nanosensor PPP-NCHR) with the spring constant of *k* ≈ 42 N m^−1^ and tip radius < 10 nm was used. The resolution of the images was set to 256 pixels × 256 pixels. The recorded images were flattened to remove the artefacts caused by sample tilt and scanner blow. The surface roughness was determined as the root-mean-square value (*R*_q_) using the WSxM 5.0 software.

Contact angles were measured using the sessile drop method with an optical tensiometer (Theta Lite, Biolin Scientific) apparatus at an ambient temperature and humidity. A drop of PBS (5 μl) was positioned on the coated substrates and was photographed immediately after positioning. The images of the drops were processed by the image analysis system, which calculated the contact angles from the shapes of the drops. To achieve a precise value, five measurements were collected for each substrate at different points on each sample. The uncoated Si/SiO_2_ substrate was considered as the control.

For the immersion test for weight loss measurements, the coated samples Ti–EG and Ti–EG-a were immersed in a PBS solution at 37 °C in an oven for 7 days. The uncoated screw-like AZ31 was considered as the control. Afterwards, the samples were ultrasonically cleaned with de-ionized water for 2 minutes, rinsed with de-ionized water three times, and blow-dried. The percentage of mass was calculated using eqn (1):1
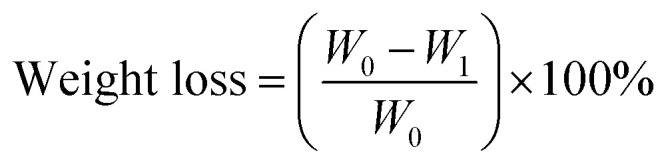
*W*_0_ and *W*_1_ are the mass of the samples before and after immersion. The surface morphology of the degraded samples was characterized using SEM.

The pH value of the PBS solution before and after the immersion test was measured using a standard laboratory pH meter (Thermo Scientific, Orion Star A211) at 22.3 °C. The biodegradation products after immersion in PBS were also analyzed using powder X-ray diffraction (PXRD) and scanning transmission electron microscopy (STEM). The calcium (Ca) content in the uncoated AZ31 alloy acquired from Goodfellow was determined using inductively coupled plasma optical emission spectrometry (ICP-OES, Jobin Yvon Activa M). Uncoated AZ31 shavings from the cutting process were used for the ICP-OES analysis.

### 2.4. Short-term cytotoxicity

Cytotoxicity analysis was conducted following the protocol described by Costa *et al.*^32^ The specimens were incubated with the minimum essential medium (MEM) for 24 hours, and then the medium was collected and filtered using a 0.45 μm pore-size filter. Throughout these tests, the material-to-fluid extraction ratio was kept constant at 0.2 g ml^−1^. The latex extracts prepared with the same extraction protocol were used as positive controls for cell death. L929 rat lung fibroblasts (from the European Collection of Cell Cultures) were seeded in 24-well plates (*n* = 3, 5 × 10^3^ cells per well) and incubated at 37 °C in a humidified atmosphere with 5% CO_2_ for 24 hours. The cells were cultured in Dulbecco's Modified Eagle Medium (DMEM) supplemented with 10% fetal bovine serum (Gibco, Barcelona, Spain) and 1% antibiotic–antimycotic solution (Sigma). After 24 hours, the culture medium was removed from each well and replaced with the MEM extraction fluid, followed by an additional 72 hour incubation at 37 °C in a humidified 5% CO_2_ environment. A live/dead assay was then performed by staining the live cells with calcein-AM (1 mg ml^−1^; Molecular Probes, Eugene, OR) and nonviable cells with propidium iodide (0.1 mg ml; Molecular Probes). Fluorescent images were captured using a fluorescence microscope (BX-61; Olympus, Hamburg, Germany) to assess the cell viability.

## 3. Results and discussion

### 3.1. Ti–EG ALD/MLD on Si/SiO_2_ substrates

**Fig. 3:**
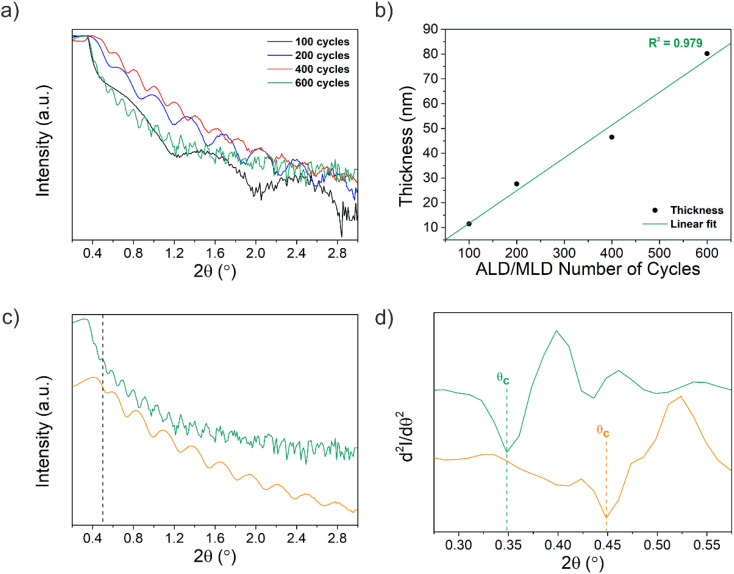
(a) XRR patterns of Ti–EG on Si/SiO_2_ substrates after 100, 200, 400 and 600 ALD/MLD cycles prepared at 105 °C and (b) thickness as a function of the number of cycles. (c) XRR patterns of the as-deposited (green line) Ti–EG and air-annealed Ti–EG-a (orange line) thin films deposited on Si/SiO_2_ substrates grown at 105 °C after 600 ALD/MLD cycles. The patterns are shifted along the *y*-axis for clarity of presentation; a dashed line is added as a guide to the eye to highlight the XRR pattern differences between the as-deposited and annealed films. (d) Second derivative for *θ*_c_ estimation for both samples.

A general ALD/MLD process is performed within a suitable temperature range, which depends on the precursor's reactivity and stability. Thus, the deposition temperature plays an important role in the ALD/MLD process, as the thin film properties strongly depend on this parameter. The deposition temperature of 105 °C was chosen as the lower limit to avoid the condensation of EG that requires an evaporation temperature of 90 °C and thermal decomposition in the gas phase or at the substrate surface. This deposition temperature provided enough thermal energy to drive a complete reaction between the TTIP and EG, as a bi-functional organic alcohol, to form the (–Ti–O–CH_2_–CH_2_–O–Ti–; Ti–EG) hybrid thin films on Si/SiO_2_ substrates, allowing the investigation of the Ti–EG growth evolution, before the ALD/MLD coating of the AZ31 screw-like substrates. The ALD/MLD hybrid films using various number of cycles were characterized using XRR to determine their thickness. The relationship between film thickness and the number of ALD/MLD cycles at 105 °C is shown in Fig. 3a. This is, in fact, an important characteristic expected for an ideal ALD/MLD process. The plot of the thickness of Ti–EG as a function of the number of ALD/MLD cycles represents good linearity (*R*^2^ = 0.979) with a slope pointing to a growth per cycle (GPC) of 0.13 nm per cycle (Fig. 3b).

The process parameters were optimized for film uniformity and conformality and not for the growth per cycle value, and therefore, this value can probably still be improved. In fact, previous research has shown higher growth per cycle values in a deposition temperature range of 90–115 °C.^33^ To this end, the conformality of the deposition was studied using STEM at different image modes on high aspect ratio nanostructured substrates. Supported carbon nanotube forests were used as a prototypical representative of this class of materials. The STEM studies confirmed that the Ti–EG coating is conformal and uniform, further demonstrating that the Ti–EG grows under a surface-controlled deposition (Fig. S3†). The Si/SiO_2_ sample coated with 600 Ti–EG ALD/MLD cycles was chosen for further studies; the as-deposited Ti–EG and air-annealed Ti–EG-a samples were analyzed using XRR and the thickness and mass density (*ρ*_m_) of both films were extracted. Fig. 3c shows the XRR patterns for the Ti–EG and air-annealed Ti–EG-a films, where the periodicity depends on the thickness, and the thicker the film, the shorter the period of the Kiessig fringes. Therefore, the change in the Kiessig fringe shape is a consequence of the annealing step, causing a volume shrinkage of the film, as confirmed by comparing the thickness values. The thickness of the Ti–EG-a sample (36.8 nm) is indeed ∼54% lower than that of the Ti–EG sample (80.2 nm).

The second derivative of the XRR data to determine the critical angle value (*θ*_c_) is presented in Fig. 3d, S4 and Table S1,† and it shifts to higher angles upon the annealing step. From the *θ*_c_ values, the estimated density of the Ti–EG was 1.4 g cm^−3^, which is much less than the density of 2.5 g cm^−3^ for the air-annealed Ti–EG-a (more details on the density determination are given in the ESI†). This result highlights the difference in the chemical nature of the two films.

**Fig. 4:**
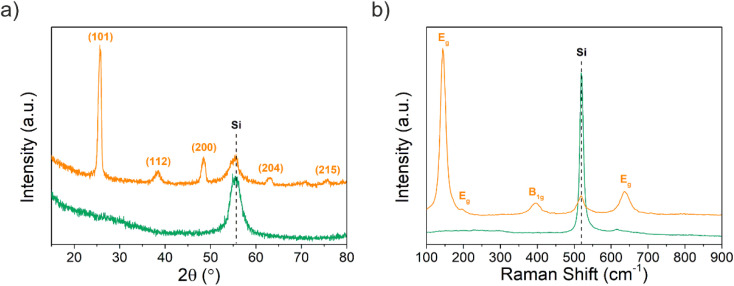
(a) GIXRD pattern and (b) Raman spectra of the as-deposited (green line) Ti–EG and air-annealed Ti–EG-a (orange line) thin films deposited on Si/SiO_2_ substrates grown at 105 °C after 600 ALD/MLD cycles. The observed broad reflection at 50–60° is assigned to the characteristic (311) plane of the crystalline Si(100) substrate.

Grazing incidence X-ray diffraction (GIXRD) was performed to measure the structural characteristics of the deposited Ti–EG and the air-annealed Ti–EG-a on Si/SiO_2_ substrates, and the results are given in Fig. 4a. No obvious diffraction peak is observed for Ti–EG, except one peak arising from the Si/SiO_2_ substrate. In contrast to Ti–EG, the air-annealed Ti–EG-a exhibits diffraction peaks that are all attributed to anatase TiO_2_, according to the anatase TiO_2_ phase reference reflections corresponding to JCPDS card no. 01-083-5914, which is depicted in Fig. S5.† The estimated lattice parameters of the sample considered are listed in Table S2,† which matches the literature values.

Fig. 4b shows the Raman spectra, and according to the factor group analysis, anatase TiO_2_ has six Raman active modes (A_1g_ + 2B_1g_ + 3E_g_), three infrared active modes (A_2u_ + 2E_g_) and one vibration of B_2u_, which is inactive in both infrared and Raman spectra.^34^ TiO_2_ in an anatase phase shows five typical Raman modes: E_g_ (143 cm^−1^), E_g_ (196 cm^−1^), B_1g_ (397 cm^−1^), A_1g_ (517 cm^−1^) and E_g_ (637 cm^−1^).^35,36^ As shown in Fig. 4b, the strongest band at 143.9 cm^−1^ belongs to the Ti–O bending of the υ6 (E_g_) mode, and a small peak at 197.1 cm^−1^ represents intrinsically weak vibrations (E_g_ mode) in anatase TiO_2_ on the Si/SiO_2_ substrate after the annealing step. In addition, the characteristic bands at 396.1, 518.7, and 636.7 cm^−1^ were attributed to the B_1g_, B_1g_ + A_1g_, and E_g_ modes, respectively. There is no characteristic Raman band belonging to TiO_2_ in the Ti–EG sample, which indicates that the Ti–EG film is dominated by an amorphous phase. However, the Ti–EG-a sample, which is obtained by further annealing Ti–EG at 400 °C for 4 hours in the air, shows the characteristic bands of anatase TiO_2_, consistent with the GIXRD results.

**Fig. 5:**
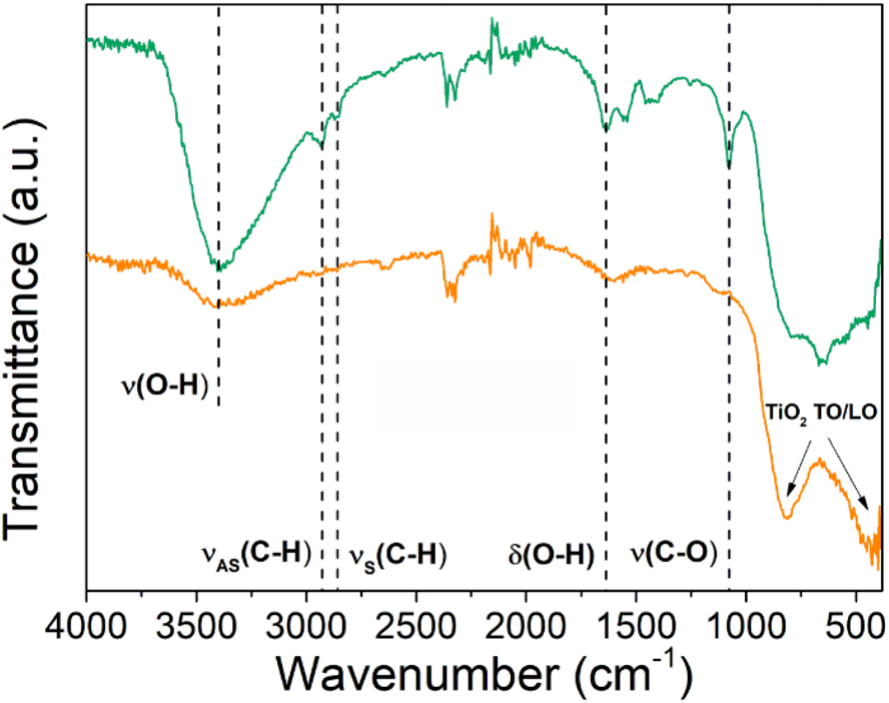
ATR-FTIR spectra of the as-deposited (green line) Ti–EG and air-annealed Ti–EG-a (orange line) thin films deposited on Si/SiO_2_ substrates grown at 105 °C after 600 ALD/MLD cycles.

The influence of the annealing treatment step on the chemical bonding structure of the Ti–EG hybrid films was studied using attenuated total reflectance Fourier transform (ATR-FTIR), as shown in Fig. 5. The ATR-FTIR analysis of the Ti–EG sample provides information on the organic moieties in the films and the resulting chemical bonding structure. For instance, the peaks at 2933 cm^−1^ and 2865 cm^−1^ can be ascribed to the asymmetrical (*ν*_AS_) and symmetrical (*ν*_S_) CH_2_ stretching vibration modes, respectively. In addition, the C–O stretching vibration peak is located at 1082 cm^−1^, confirming the presence of the aliphatic organic diol from the EG.^37^ After the annealing step, the peaks located at around 1082 cm^−1^ and 1641 cm^−1^ disappeared, suggesting that the organic moieties from the EG in the ALD/MLD film were removed and the hybrid Ti–EG was converted to TiO_2_. This finding is also supported by the vibrational modes at around 432.1 cm^−1^ and 817.8 cm^−1^ assigned to the longitudinal and transverse optical (LO and TO, respectively) phonon modes of TiO_2_, respectively.^38^

**Fig. 6:**
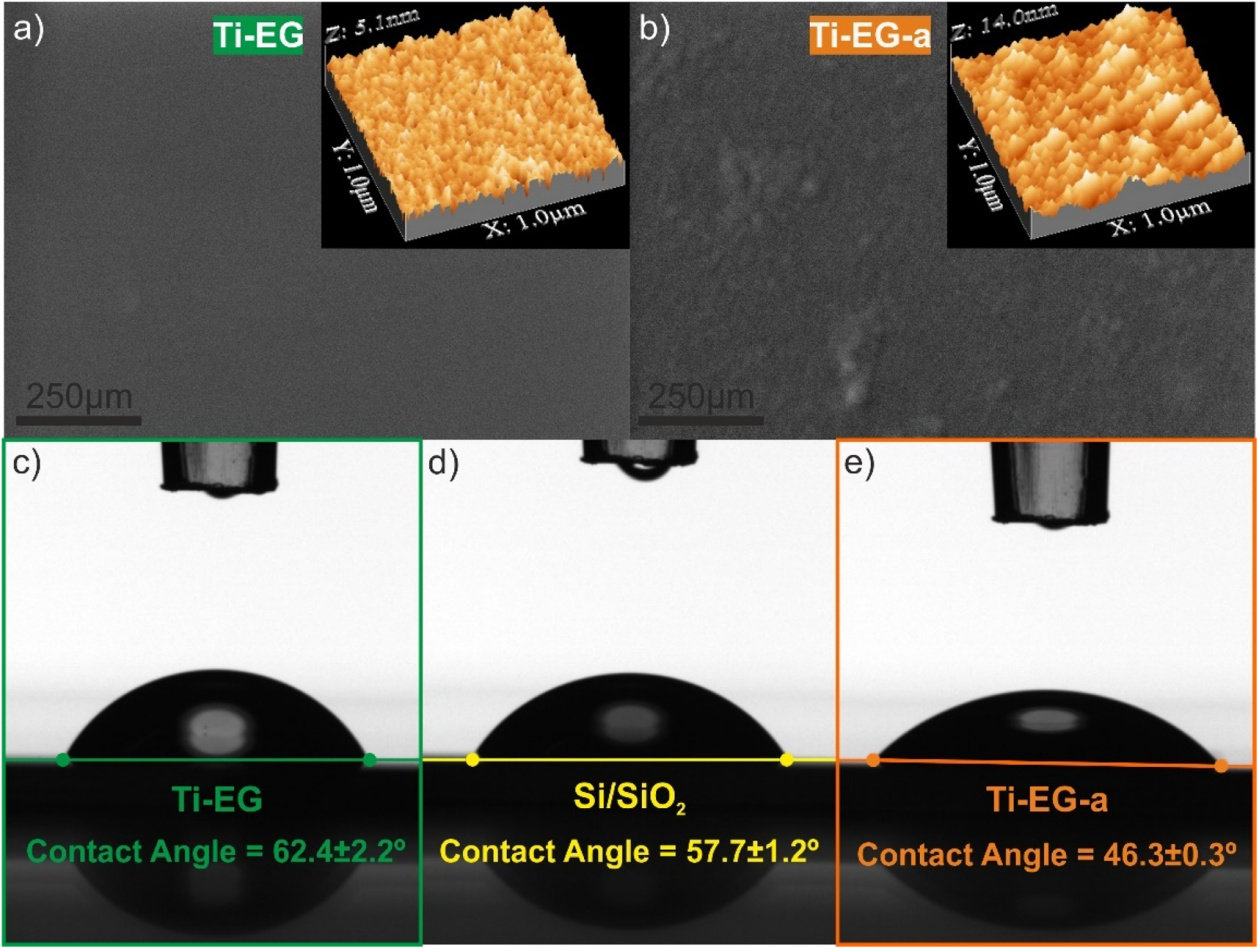
Top-view SEM images of (a and b) as-deposited Ti–EG and (c and d) air-annealed Ti–EG-a thin films deposited on Si/SiO_2_. The inset in (a and b) depicts 3D rendered AFM topography images (1 μm × 1 μm) of both thin films. Contact angles on: (c) Ti–EG thin film on Si/SiO_2_; (d) bare Si/SiO_2_ substrate and (e) air-annealed Ti–EG-a thin film on Si/SiO_2_. Uncoated Si/SiO_2_ substrate was considered as a control. (5 μl of PBS droplets on different substrates).

The surface morphology of the thin films is shown in Fig. 6a and b. The Ti–EG thin film appears to be featureless, as expected for an amorphous film (Fig. 6a). On the other hand, some particulates can be seen on the surface of the Ti–EG-a thin film (Fig. 6b). No cracks were observed on the surface of both films. Additionally, the inset in Fig. 6a and b shows the surface topography of the Ti–EG and Ti–EG-a thin films, respectively. The Ti–EG thin film exhibits low surface roughness, with a root-mean-square (*R*_q_) roughness of 0.56 nm, while for the Ti–EG-a thin film, the surface roughness value was slightly higher, and it was found to be 1.19 nm. This roughness change suggests phase transformation. These values were ascertained from a 1 μm × 1 μm image (Fig. S6†).

The wettability of Ti–EG and Ti–EG-a thin films deposited on Si/SiO_2_ substrates compared with uncoated Si/SiO_2_ substrates was measured using static water contact angle (WCA) measurements. Taking into consideration the envisioned application of the hybrid films, the water was replaced with a PBS solution. The uncoated Si/SiO_2_ substrate showed a contact angle of 57.7° ± 1.2°, indicating that the surface is hydrophilic (a surface is hydrophilic if WCA < 90°), as shown in Fig. 6c–e. The Ti–EG-a sample (46.3 ± 0.3) exhibits a more hydrophilic characteristic than Ti–EG (62.4 ± 2.2), as the PBS droplet was not able to fully spread on the Ti–EG surface. Annealing the Ti–EG sample influences the film strongly and it becomes more hydrophilic as the contact angle decreases while the surface roughness increases, establishing a relationship between wettability and surface roughness. In our previous work,^19^ we grew anatase TiO_2_ by ALD using TTIP and deionized water (H_2_O) at 200 °C, and it was found to be highly hydrophilic, suggesting that inorganic TiO_2_ is inherently more hydrophilic than titanium-based hybrid coatings. Moreover, the wettability properties of the TiO_2_ ALD coating on Ti alloy substrates were favorable for the enhancement of cell proliferation activity, where the TiO_2_ surfaces were moderately hydrophilic, *i.e.*, WCA values ranging from 60° to 80°.^39^ The authors claimed that this enhancement could be triggered by a combination of changing the chemistry and wettability of the Ti alloy surface with the TiO_2_ ALD coating. In this context, it is expected that coating a hydrophilic Ti–EG film onto the AZ31 screw-like substrates will be beneficial for biological activity while providing physical protection.

### 3.2. Ti–EG ALD/MLD on AZ31 screw-like substrates

After studying the ALD/MLD process parameters on the Si/SiO_2_ substrates, the ALD/MLD process was applied to the AZ31 screw-like substrates, and they were coated using 600 ALD/MLD cycles.

**Fig. 7:**
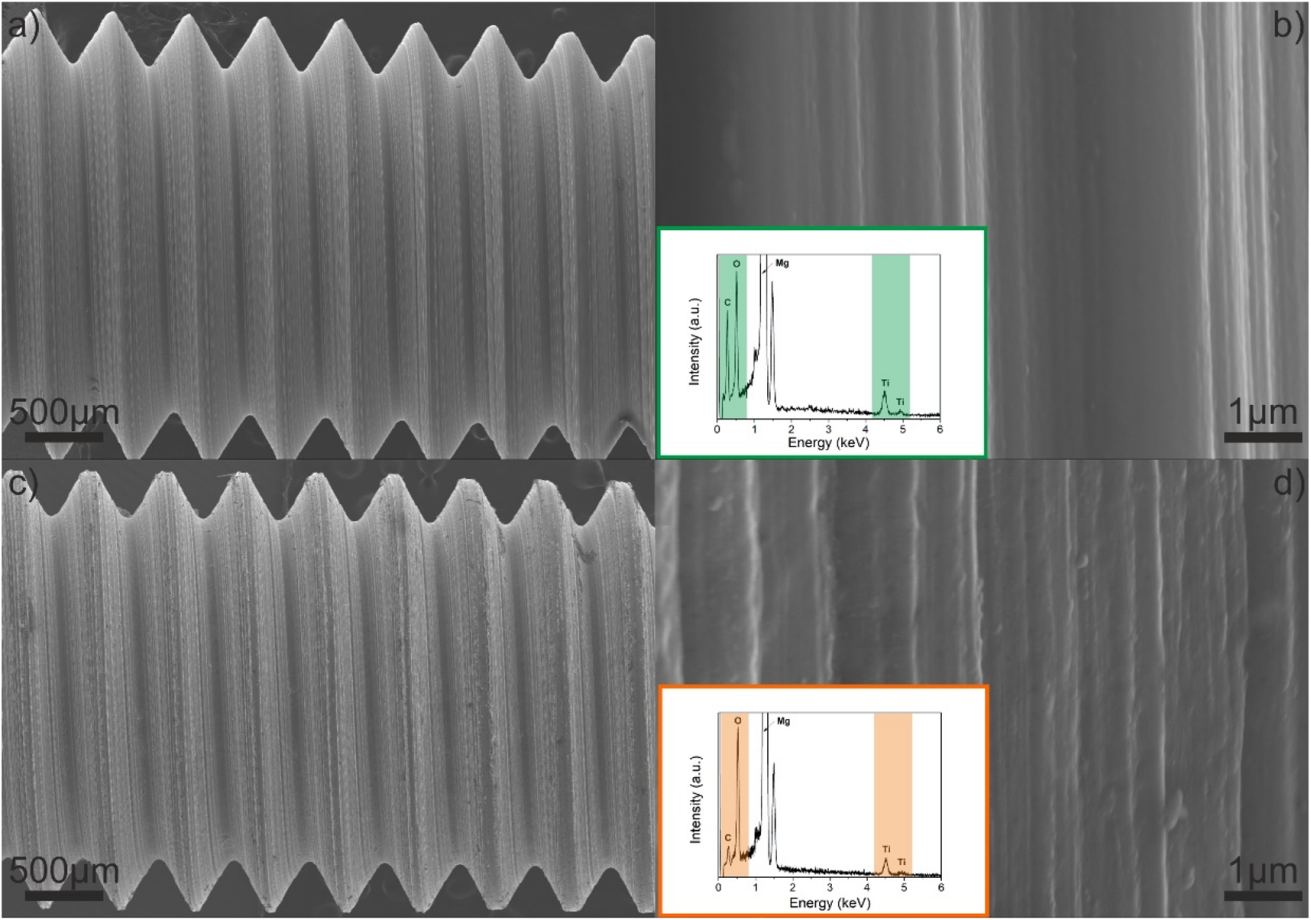
Top-view SEM images of (a and b) as-deposited Ti–EG and (c and d) air-annealed Ti–EG-a thin films deposited on the AZ31 screw-like substrate grown at 105 °C after 600 ALD/MLD cycles. The insets reveal the EDX spectra.

Low magnification top-view SEM images of both Ti–EG and air-annealed Ti–EG-a thin films deposited on the AZ31 screw-like substrates are shown in Fig. 7a and c and their surfaces appear very clean. As can be seen from Fig. 7b and c, the high magnification top-view SEM images of both Ti–EG and air-annealed Ti–EG-a thin films are smooth without any apparent surface defects, and the corresponding EDX spectra (inset figures and Fig. S7†) acquired at this magnification, between two threads, further revealing the presence of the Ti element on the AZ31 screw-like surfaces.

**Fig. 8:**
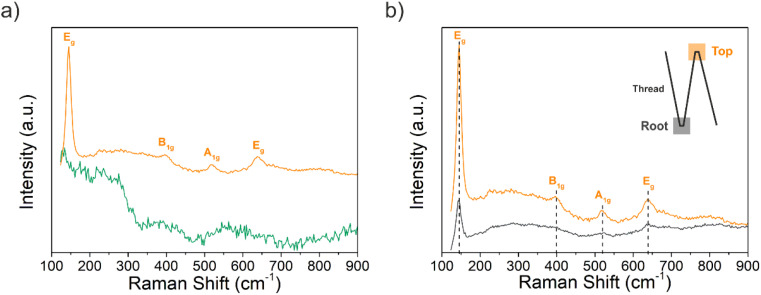
(a) Raman spectra of the as-deposited (green line) Ti–EG and air-annealed Ti–EG-a (orange line) thin films deposited on AZ31 screw-like substrates grown at 105 °C after 600 ALD/MLD cycles. (b) Raman spectra of the air-annealed Ti–EG-a thin film were recorded in two different regions of the AZ31 screw-like thread, *i.e.*, orange line – top and gray line – root (inset-schematic illustration).

Following morphological and elemental analysis of both Ti–EG and Ti–EG-a thin films, their structure was probed by Raman spectroscopy, as displayed in Fig. 8a. The experimental Raman spectrum of the Ti–EG-a thin film displays the typical fingerprint of anatase TiO_2_, showing four main bands located at 144.8 cm^−1^ (E_g_ mode), 396.1 cm^−1^ (B_1g_ mode), 516.4 cm^−1^ (A_1g_ modes), and 637.5 cm^−1^ (E_g_ mode), corresponding to the O–Ti–O bending and Ti–O stretching modes. The band located at 516.4 cm^−1^ in this sample does not overlap with the Raman band of the Si/SiO_2_ substrate. Moreover, Raman spectroscopy has been proven to be efficient in discriminating the anatase TiO_2_ phase onto the AZ31 screw-like surface, matching the previous results on Si/SiO_2_ substrates. Fig. 8b depicts the Raman spectra of the Ti–EG-a thin film acquired on two regions of the thread by focusing a laser beam on the top and root region of the thread (inset of Fig. 8b). It is safe to assume that the Ti–EG-a maintained its uniformity throughout the AZ31 screw-like threads, supported by the detected modes attributed to crystalline TiO_2_. Based on this detailed analysis, it could be concluded that the Ti–EG films were successfully prepared on AZ31 screw-like substrates through ALD/MLD and further transformed into anatase TiO_2_ (Ti–EG-a) by the post-annealing treatment step.

**Fig. 9:**
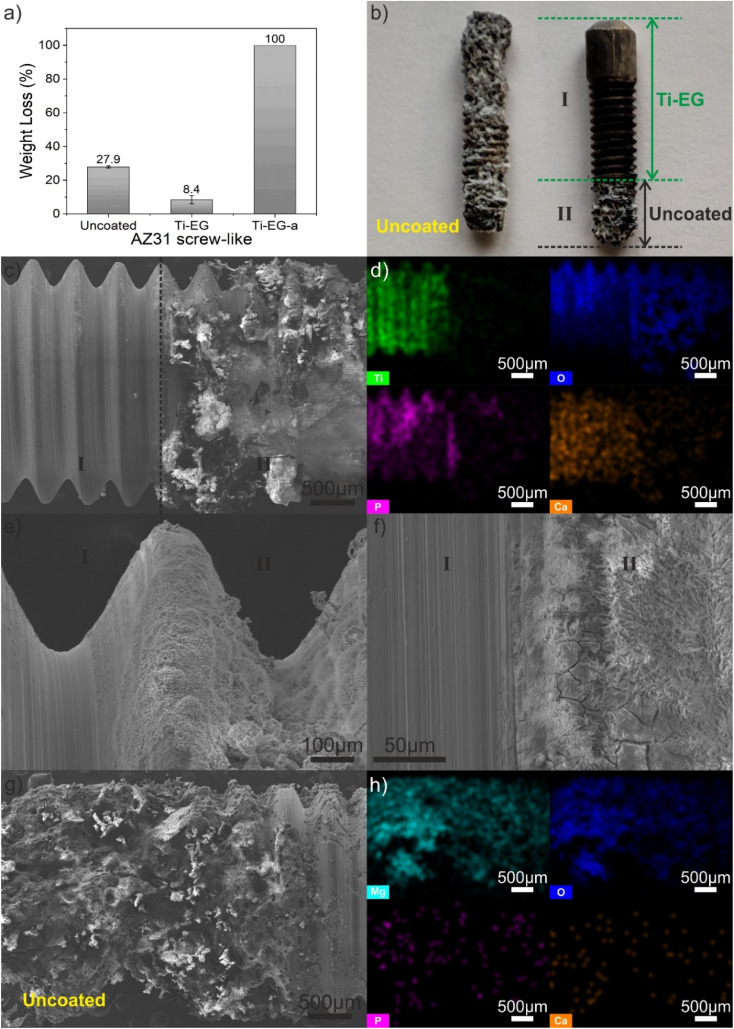
(a) Weight loss percentage after PBS solution immersion at 37 °C for 7 days, (b) digital camera photograph of an uncoated (control) and partially coated AZ31 screw-like substrates after PBS immersion for 7 days, (c) top-view low-magnified SEM image and (d) corresponding EDX elemental mapping, (e and f) top-view high-magnified SEM images of (c). (g) Top-view low-magnified SEM image and (h) corresponding EDX elemental mapping of the uncoated (control). The weight loss was calculated using the weight, including the white degradation layer. The error bars indicate mean ± standard deviation.

From the above characterizations, it is evident that the Ti–EG thin film was deposited on the surface of the AZ31 screw-like substrate. The film, consisting of amorphous Ti–EG and crystalline Ti–EG-a, covers the surface with excellent uniformity and attaches well to the AZ31 screw-like substrates. In our study, we have chosen a short-term weight loss assay as a selection criterion between the amorphous (Ti–EG) and crystalline (Ti–EG-a) samples for bioactivity surface tests. To this end, the samples were immersed in a PBS solution at 37 °C for 7 days and the AZ31 screw-like substrates were partially coated with Ti–EG and the corresponding annealed Ti–EG-a thin films in order to highlight their protective behavior. Fig. 9a shows the mean degradation percentage determined using eqn (1) after the immersion test. The highest weight loss percentage corresponds to Ti–EG-a, while Ti–EG shows the lowest weight loss percentage when compared to the uncoated sample.

The total loss of the AZ31 screw-like substrate partially coated with the Ti–EG-a sample can be explained by considering, first, the impact of the air annealing step on the AZ31 alloy and then on the Ti–EG-a film. It has been demonstrated that the heat treatment of the AZ31 alloy changes its microstructure, which, in turn, improves the degradation behavior of the alloy. However, it does not adequately solve the problem of rapid degradation.^40^ In addition, our air annealing step also introduced changes in the AZ31 alloy supported by XRD analysis by monitoring the FWHM values of three main XRD peaks, as shown in Fig. S8 and Table S4.† The lowest FWHM value calculated from the second diffraction was considered as a structural indicator. On the other hand, the air annealing step transformed the Ti–EG into anatase TiO_2_ (Ti–EG-a), which can induce a certain degree of porosity formation as the organic part of the hybrid is removed by oxidizing in air at 400 °C for 4 hours, together with the decrease in thickness, as previously reported in the literature.^41–44^ Briefly, these features may reduce the physical protection capability of Ti–EG-a on the AZ31 screw-like substrate surface when immersed in the PBS medium.

**Fig. 10:**
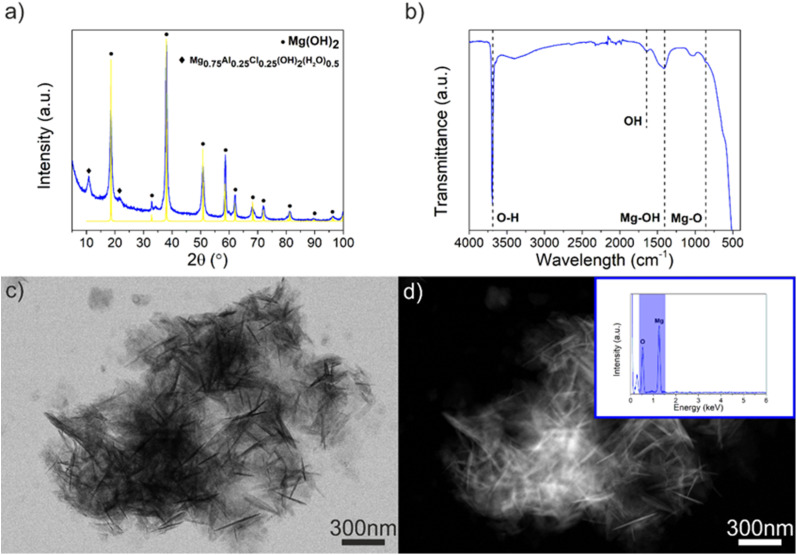
(a) Powder XRD pattern and (b) ATR-FTIR spectrum (c) TE-STEM mode image and (d) the corresponding ZC-STEM mode image of the degradation products (Mg(OH)_2_). The inset in (d) reveals the EDX spectrum, confirming the presence of O and Mg.

Fig. 9b shows the digital camera photograph of a partially coated AZ31 screw-like substrate showing the effect of PBS immersion on the surface morphology. A transformation of the screw-like threads from a smooth to a rough surface of the uncoated section and the noticeable color change from dark silver to white is observed (Fig. 9b). This finding is also supported by Fig. 9c, where two different regions are distinctly observed: region II is clearly transformed with irregular features and the opposite region I remains unchanged. EDX elemental mapping (Fig. 9d) further reveals the presence of Ti throughout the coated region. Interestingly, the EDX mapping also shows the existence of Ca and P, especially on the coated region, which could be associated with the physical deposition of Ca^2+^ and PO_4_^3−^ on the surface.^45^ This finding can be related to the presence of surface hydroxyl groups (–OH) from the Ti–EG hybrid film, supported by the ATR-FTIR results in Fig. 5, which implies that the –OH groups will attract Ca^2+^ ions, and in the next step, the PO_4_^3−^ ion binds the Ca^2+^ ions, in contrast to the uncoated AZ31 screw-like (control) substrate, with no Ca^2+^ and PO_4_^3−^ on its surface (Fig. 9g, h and S9†). Based on the presented results, it is important to identify the origin of Ca, which can be directly related to the AZ31 chemical composition and released during the PBS immersion. As confirmed by the ICP-OES analysis of uncoated AZ31 shavings from the cutting process, the Ca content was determined to be 0.24 mg g^−1^, besides the other alloying elements (*e.g.*, Al, Zn). On the other hand, the AZ31 screw-like sample coated with Ti–EG immersed for 7 days in the PBS medium was analyzed using Raman spectroscopy. As an example, Fig. S10† shows the spectrum acquired from the AZ31 coated sample, exhibiting a broad band at 971 cm^−1^, which can be attributed to the PO_4_^3−^ on the surface of the coated sample (region I). In accordance with ref. 46, the frequency region of the PO_4_^3−^ ions is located between 930 and 980 cm^−1^. Fig. 9e and f reveals the SEM images at different magnifications at the interface between the coated and uncoated regions. As demonstrated in Fig. 9e, the Ti–EG thin film is as smooth as that of the same sample before immersion (Fig. 7a). On the other hand, the surface of region II is covered with a layer comprised of flower-like structures and microcracks (Fig. S11†). After the immersion test, the degradation products were collected for further characterizations, and these results are given in Fig. 10.

The powder XRD peaks marked by black dots were indexed to the hexagonal structure for magnesium hydroxide (Mg(OH)_2_) according to JCPDS card 04-016-4785 (yellow pattern). The two small peaks marked by black rhombus were indexed to magnesium aluminum chloride hydroxide hydrate (Mg_0.75_Al_0.25_Cl_0.25_(OH)_2_(H_2_O)_0.5_), compatible with JCPDS card 04-024-7291 (Fig. 10a). The ATR-FTIR spectrum has a sharp and intense peak at 3697.3 cm^−1^ (Fig. 10b), and it is attributed to the O–H band stretching in the Mg(OH)_2_ structure.^47^ The TE-STEM mode image and the corresponding ZC-STEM mode image are shown in Fig. S10c and d† and they reveal the Mg(OH)_2_ structure. It can be concluded that the magnesium hydroxide (brucite, Mg(OH)_2_) was the main degradation product. As reported previously, Mg(OH)_2_ is one of the commonly formed products during the degradation process,^48^ which leads to an increase in the medium pH. In fact, the pH measurements of the medium obtained after the immersion test revealed that the uncoated sample produced a slightly higher pH value (11.4) than Ti–EG (10.8) and Ti–EG-a (10.9), considering the initial pH value of 7.5 from the PBS. This phenomenon is related to the production of hydroxide ions (OH^−^) and hydrogen (H_2_) as a result of the water (H_2_O) reduction reaction supporting the anodic dissolution of Mg.^49^ Consequently, the pH level can be shifted to the alkaline region.^48^ In addition to the time-dependent weight loss assay, electrochemical impedance spectroscopy and potentiodynamic tests must be conducted in future studies.

### 3.3. Cytotoxicity of Ti–EG ALD/MLD on AZ31 screw-like substrates

**Fig. 11:**
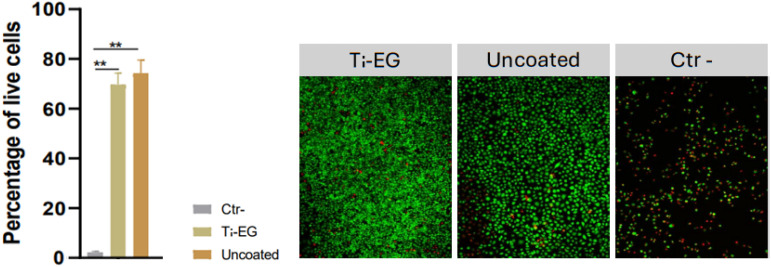
Viability of L929 cells after incubation with MEM extraction fluid. Representative fluorescence images for each of the three conditions, showing live cells stained with calcein-AM (green) and nonviable cells stained with propidium iodide (red). Statistical analysis was performed using one-way ANOVA, followed by *post hoc* Bonferroni testing for group comparisons. Data are presented as mean ± SD. *N* = 3, **p < 0.01.

The cytotoxicity of Ti–EG ALD/MLD on AZ31 screw-like substrates was evaluated by comparing it with uncoated AZ31 screw-like substrates. This assessment aimed to determine the potential toxic effects of substances released from the metallic scaffolds after a 24 hour incubation with a standard culture medium (MEM). The MEM extraction fluid was then applied to the L929 cells and incubated for three days. The cell viability was determined by staining live cells with calcein-AM and nonviable cells with propidium iodide. Fig. 11 shows that the percentage of live cells, both with or without coating, was statistically similar and significantly different when compared to the negative control.

The cytotoxicity results indicate that the Ti–EG ALD/MLD coating did not introduce any significant toxic effects compared to the uncoated AZ31 screw-like substrates. The comparable percentage of live cells in both coated and uncoated samples suggests that the Ti–EG ALD/MLD coating does not release harmful substances during the 24 hour incubation period in MEM. This outcome is promising for potential biomedical applications, as Ti–EG ALD/MLD on AZ31 may offer a viable approach for enhancing substrate stability or functionality without compromising biocompatibility, though further long-term studies would be beneficial for confirming these findings in extended *in vivo* conditions as well as mechanical features of the Ti–EG ALD/MLD hybrid coating.

## 4. Conclusion

In this work, a well-functioning ALD/MLD process was developed, which yielded a uniform deposition of Ti-based hybrid films (Ti–EG) on complex structures such as the AZ31 screw-like substrates. The results indicated that the hybrid coating of Ti–EG on the AZ31 screw-like substrate improved its physical and biodegradation behaviors. The as-deposited hybrid thin films were amorphous and hydrophilic and the bonding structure was analyzed using ATR-FTIR, which confirmed the hybrid coating formation. On the other hand, Raman spectroscopy was used to determine the structure of the deposited thin films as well as the physical deposition of the phosphate ion on the coated AZ31 screw-like surface. The weight loss data provided guidance and served as a reference for the application of the AZ31 alloy in physiological environments, where the test can differentiate between the coated and uncoated AZ31 screw-like substrates. In conclusion, our findings suggest that the degradation resistance was microstructure-dependent, as confirmed by the weight loss test. Additionally, the Ti–EG hybrid film showed no cytotoxicity, which is a crucial feature for biomedical applications. We expect that these positive results could serve as a strong motivation for further ALD/MLD process developments for other metallic or alloy materials and future implantable systems.

## Data availability

It includes additional experimental details and descriptions of the materials, substrates, characterization, conformality test and methods, complementary characterization, STEM images, EDX mapping, GIXRD patterns and Raman spectrum.

## Author contributions

R. M. Silva: writing–review & editing, writing–original draft, methodology, investigation, formal analysis, and conceptualization. F. J. Oliveira: writing–review & editing, methodology, investigation, formal analysis, conceptualization, and funding. M. F. Lima: review & editing, methodology, investigation, formal analysis, and conceptualization. N. A. Silva: review & editing, methodology, investigation, formal analysis, conceptualization, and funding. G. Miranda: writing–review & editing, methodology, investigation, formal analysis, conceptualization, and funding.

## Conflicts of interest

There are no conflicts to declare.

## Supplementary Material

RA-015-D4RA07952C-s001
