# Supplementary material for: Titanium-based hybrid coatings grown using ALD/MLD onto AZ31 screw-like supports for implantable systems
Source: RSC Adv. 2025 Apr 7;15(14):10774–86. doi: 10.1039/d4ra07952c (PMC11973893; doi:10.1039/d4ra07952c)
Supplement: RA-015-D4RA07952C-s001 [file RA-015-D4RA07952C-s001.pdf]

Supplementary Information (SI) for RSC Advances.
This journal is © The Royal Society of Chemistry 2025

Supplementary Information

Titanium-based hybrid coatings grown by ALD/MLD onto AZ31 screw-like supports for implantable systems

[a]R.M. Silva*, [a]F.J. Oliveira, [b,c]M.F. Lima, [b,c]N.A. Silva, [a]G. Miranda*

[a]CICECO – Aveiro Institute of Materials, Department of Materials and Ceramic Engineering, University of Aveiro, 3810-193 Aveiro, Portugal
[b]Life and Health Sciences Research Institute (ICVS), School of Medicine, University of Minho, Campus de Gualtar, 4710-057 Braga, Portugal
[c]ICVS/3B's - PT Government Associate Laboratory, Braga/Guimarães, Portugal

*Corresponding authors: rmsilva@ua.pt; gmiranda@ua.pt

Substrates

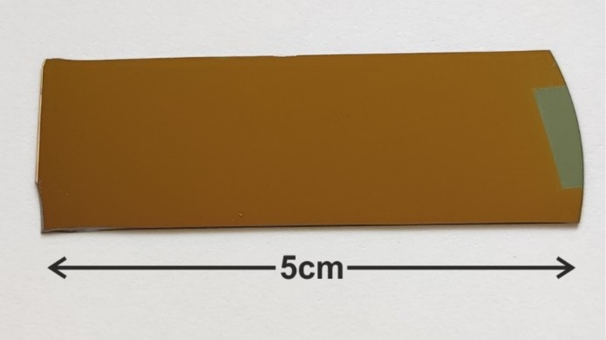

Figure S1. Digital camera photograph of a Ti-EG hybrid thin film onto Si/SiO_2 prepared at 105 ºC, after 600 ALD/MLD cycles (not to scale). A small part of the Si/SiO_2 substrate was covered with Kapton tape during the deposition to keep a portion uncoated, for comparison purpose.

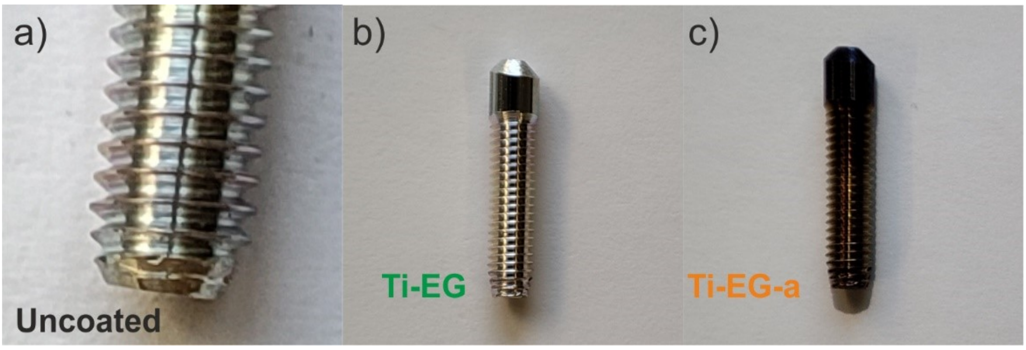

Figure S2. (a) Digital camera photograph of an AZ31 screw-like substrate (3D substrate), made from the AZ31 rod (diameter: 3 mm). It is possible to observe the tool-induced

periodic hierarchical patterns. Coated AZ31 screw-like substrates (b) before and (c) after thermal annealing step at400 ºC.

Linear film growth for Ti-EG

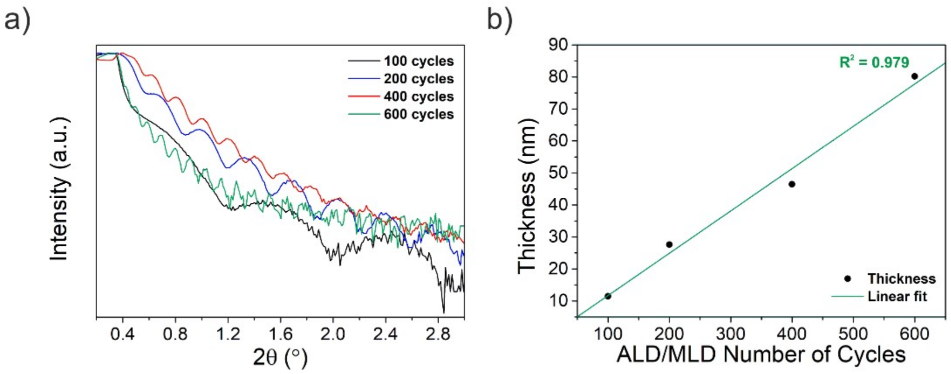

Figure S3. (a) Normalized XRR patterns of Ti-EG on Si/SiO_2 substrates after 100, 200, 400 and 600 ALD/MLD cycles prepared at 105 ºC and (b) thickness as a function of the number of cycles. Increasing the film thickness, the periodicity of the (Kiessig) fringes decreases. A linear increase of the thickness as a function of the ALD/MLD number of cycles is observed further demonstrating the self-limiting character of the process.

Conformality test

The most common way to demonstrate the conformality of an ALD process is by deposition into deep microscopic trenches (typical width <1 μm) and subsequent characterization by electron microscopy [1]. For conformality analysis, Ti-EG ALD/MLD process was also performed onto carbon nanotubes (CNTs) grown by TCVD at 650 ºC, accordingly with our previous work [2], here the CNTs were used as a test structure (high aspect ratio structure). They were coated with 200 ALD/MLD cycles of Ti-EG at 105 ºC and then imaged in a Hitachi HD2700 scanning transmission electron microscope (STEM) operated at 200 kV and equipped with energy-dispersive X-ray spectroscopy (EDX) for elemental analysis. This sample was prepared by dry adhesion of the coated CNTs to a holey carbon film supported on a copper grid.

Transmission Electron (TE)- and corresponding Z-Contrast (ZC)-STEM mode images (Figure S4b,c) provide strong evidence for good coating conformality, while EDX indicates the presence of Ti on the nanotube surface. The Ti-EG surface morphology is nicely shown in both image modes. This result also revealed, in a visual way, that the Ti-EG film was grown under surface-controlled deposition onto one dimensional (1D) CNTs.

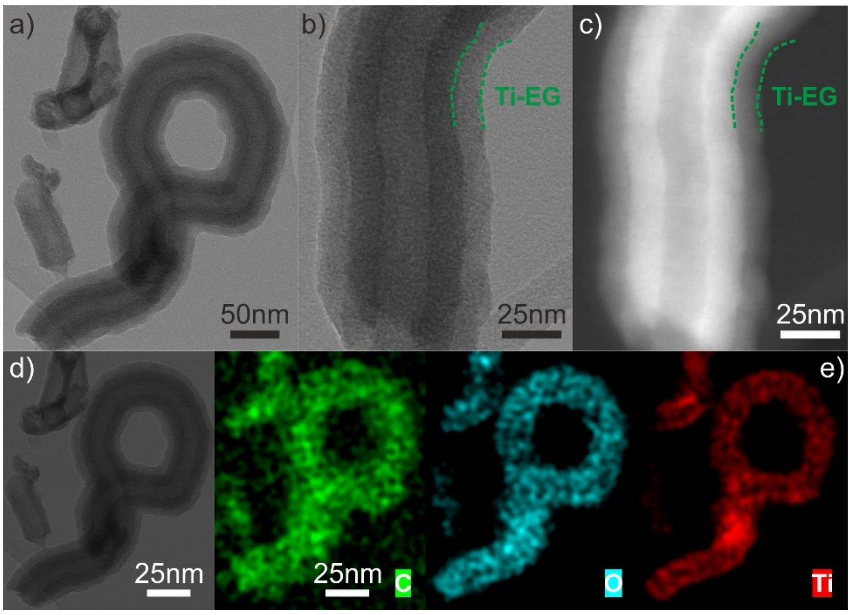

Figure S4. (a,b,d) TE-STEM mode image, (c) ZC-STEM mode image and (e) EDX elemental mapping of an individual nanotube coated with Ti-EG, with an average 13.4±0.4 nm thickness.

Density of the thin films

The non-destructive XRR technique has been widely used in thin film material characterization and belongs to the larger group of scattering methods. In addition, this technique does not rely on diffraction phenomena, which means that can be used in amorphous, crystalline and polycrystalline samples. It probes the electron density contrast with depth resolution. Once the X-rays are used to probe the electron density perpendicular to the surface. As consequence, it provides important information about thin films such as thickness (single layer and multilayers), surface roughness and density [3-5]. From the practical point of view, the technique involves measuring the reflected X-ray intensity as a function of incident angle over a range of angles closed to the critical angle (θ_c) for total reflection. The θ_c is determined as the angle at which the intensity of the XRR data drops to half of its maximum. A typical reflectivity pattern is essentially a combination of the Fresnel reflectivity and an interference pattern (Kiessig fringes) from the scattering at different layers [3-5]. The θ_c, is a material-specific property mainly dependent on film density and it is located at the first minima of the second derivative of the XRR intensity [3,6], as shown in Figure S4. The Ti-EG and Ti-EG-a thin film density (ρ_m) can be estimated from the θ_c as follows [3-5]:

$$\rho_e = \frac{\theta_c^2 \pi}{\lambda^2 r_e} (e^- \, m^{-3}) \qquad \text{Equation (1)}$$

where λ is the X-ray wavelength and r_e is the classical electron radius.

$$\rho_m = \frac{\rho_e A}{N_A Z} (g \, m^{-3}) \qquad \text{Equation (2)}$$

where A is the average of molecular weight, Z is the average of atomic number and N_A is the Avogadro constant. The electron density ρ_e may be converted to the mass density ρ_m according to Equation 2. The density (ρ_m) values were estimated and summarized in Table S1.

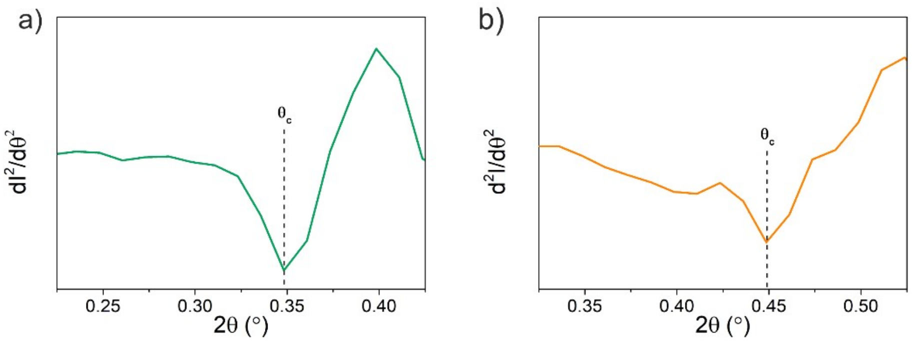

Figure S5. θ_c estimation for (a) Ti-EG after 600 ALD/MLD cycles prepared at 105 ºC and the corresponding (b) air annealed Ti-EG-a.

Table S1. Estimated density values from the XRR results.

Sample	Critical angle (θ_c)	Electron density (ρ_e)	Mass density (ρ_m)	Thickness
Ti-EG	(0.348º) 0.35º	4.4 x10^{24} e^- cm^{-3}	1.4 g cm^{-3}	80.2 nm
Ti-EG-a	(0.448º) 0.45º	7.1x10^{24} e^- cm^{-3}	2.5 g cm^{-3}	36.8 nm

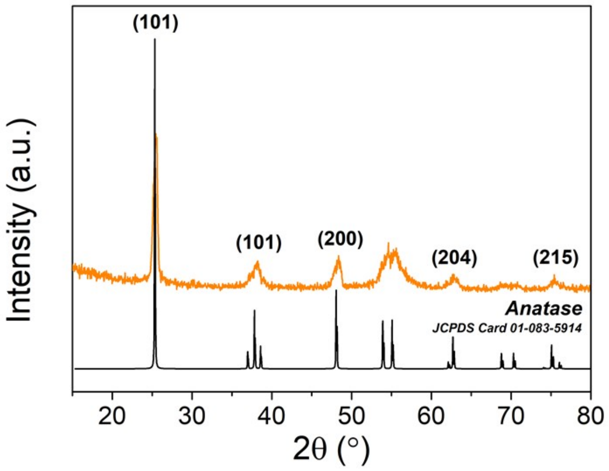

Figure S6. GIXRD patterns of Ti-EG-a and reference anatase TiO_2, confirming the anatase TiO_2 crystallographic phase. In the annealed film (Ti-EG-a), there is an obvious prominent diffraction peak at 25.39º which corresponds to the (101) crystalline plane of the TiO_2 anatase phase at 25.73º.

Table S2. Lattice constants for reference tetragonal anatase TiO_2 (JCPDS Card No. 01-083-5914) and their differences with respect to experimental measurements.

	Lattice parameters	
Reference	a/b	c
TiO_2	3.784 Å	9.511 Å
Sample	a/b	c
Ti-EG-a	3.767 Å	9.457 Å
Difference	0.449%	0.568%

The observed difference is less than 0.6 %, therefore the calculated values are in good agreement with those of the primitive anatase TiO_2 from the literature.

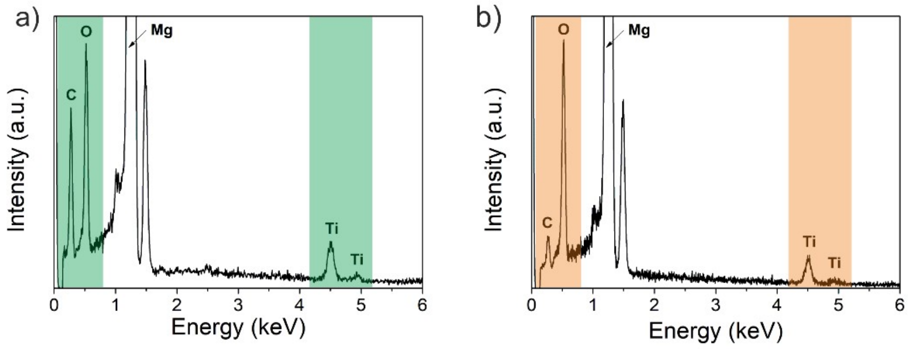

Figure S7. Normalized EDX spectra of (a) as-deposited Ti-EG and (b) air annealed Ti-EG-a thin films deposited on AZ31 screw-like substrate grown at 105 ºC, after 600 ALD/MLD cycles.

Table S3. Experimental Raman active modes of thin films, after post thermal annealing.

Samples	Ti-EG-a	Ti-EG-a
Raman shift (cm^{-1})	Si/SiO_2	AZ31 screw-like
E_g	143.95	144.83
E_g	197.16	--
B_{1g}	396.58	396.85
A_{1g}	518.73*	516.42
E_g	636.71	637.49

*This band overlaps with Raman bands of Si substrate.

The annealed thin films present the characteristic anatase TiO_2 phase Raman active modes. The observed scattering bands are well consistent with the reported literature [7].

Immersion test for weight loss measurements

XRD of AZ31 disc before and after the annealing step

The crystallographic planes of an uncoated AZ31 disc were evaluated using conventional θ-2θ diffraction in reflection mode (XRD) on a Philips X'Pert MRD diffractometer with Cu K_α (λ=1.5418 Å) radiation and a point detector. The X-rays were generated by applying 45 kV voltage on Cu anode at a current of 40 mA. The range of measurements was from 10° to 80° of the angle 2θ, with step size of 0.05º. To this end, a disc was cut from commercial extruded magnesium alloy rod (AZ31-Mg 96/Al 3/Zn 1, diameter 11 mm, Goodfellow), which matches the magnesium alloy employed in the fabrication of the AZ31 screw-like substrates (diameter 3 mm). From the practical point of view, the AZ31 disc with 11 mm of diameter allows a better sample alignment on the Philips X'Pert MRD diffractometer to perform the measurements.

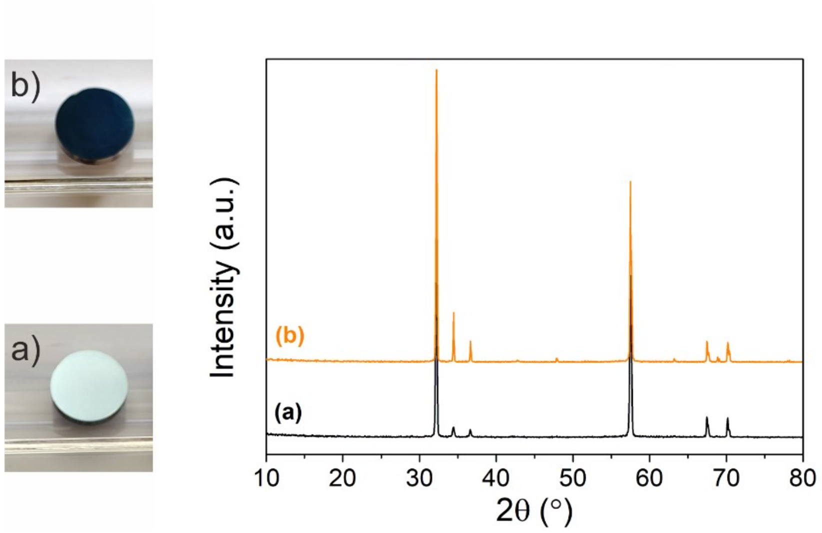

Figure S8. Digital camera photograph of uncoated AZ31 disc (diameter 11 mm); (a) as-received and (b) air annealed at 400ºC for 4 hours and corresponding XRD patterns.

In order to better evaluate the differences between the AZ31 and the air annealed AZ31-a, the 2θ position and the full width at half maximum (FWHM) of the three main XRD diffraction peaks are summarized in Table S4.

Table S4. Experimental 2θ position and corresponding FWHM values.

Samples	2θ	FWHM (º)
AZ31 (As-received)	32.19	0.19
	34.42	0.28
	36.63	0.27
AZ31-a	32.21	0.18
	34.44	0.08
	36.64	0.17

Ti-EG - uncoated region II

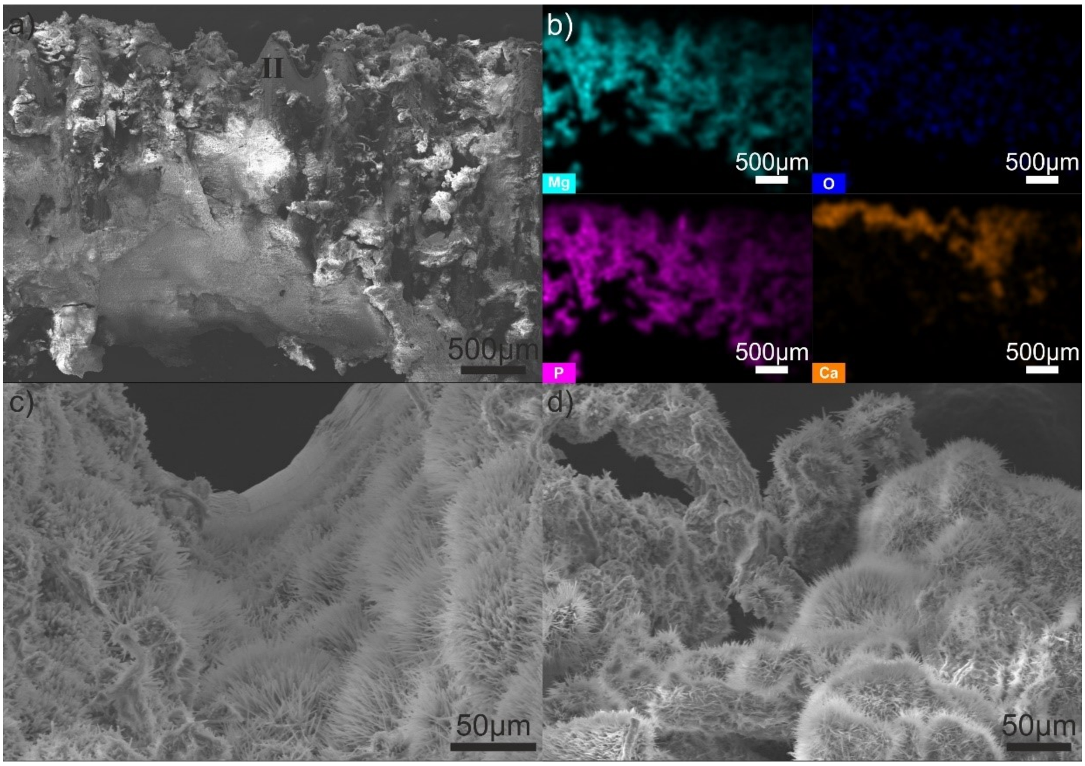

Figure S9. SEM images of the region II corresponding to the uncoated section of the partially coated AZ31 screw-like substrate. (a) Top-view low-magnified SEM image and (b) corresponding EDX elemental mapping, (c,d) top-view high-magnified SEM images of (a), exhibiting a flower-like structures.

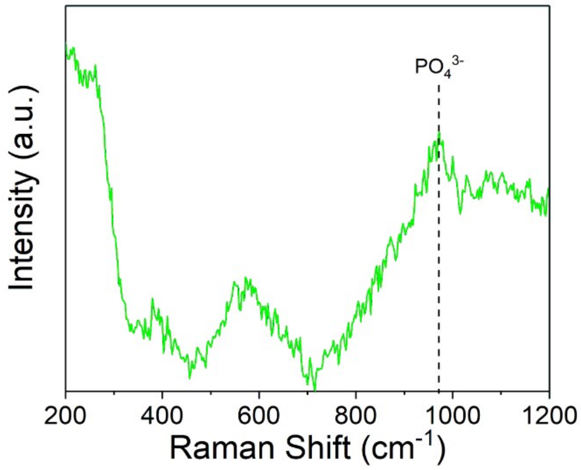

Figure S10. Raman spectrum of Ti-EG thin film deposited on AZ31 screw-like sample, after PBS immersion test.

Uncoated AZ31 screw-like (reference)

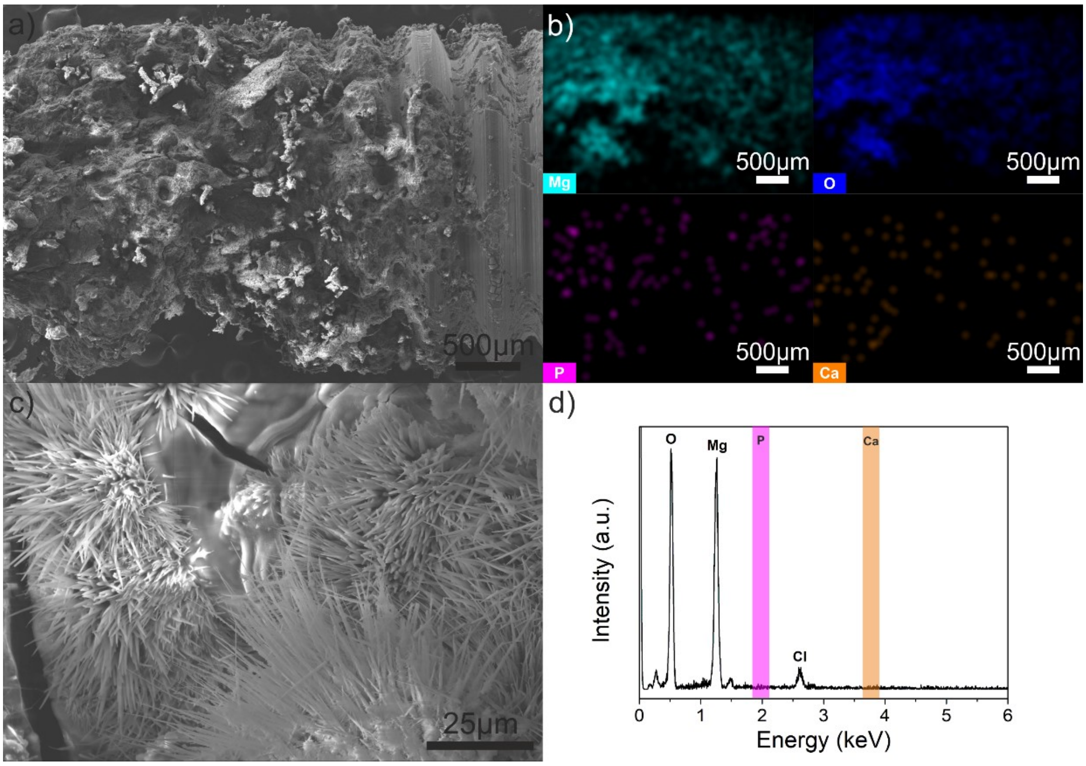

Figure S11. (a,c) Top-view SEM images corresponding (b) EDX elemental mapping and (d) spectrum of the uncoated AZ31 screw-like sample. According to the EDX spectrum, there are no peaks corresponding to P and Ca. The uncoated AZ31 screw-like surface also exhibits the flower-like structures (Mg, O).

Degradation products after immersion in PBF

The degradation products were collected by suction filtration. To do so, a Whatman filter paper was employed and then the solution was carefully and homogenously drop-cast onto the filter paper. The product left on the top was washed several times with double-distilled water dried in oven at 60 °C and used for further characterization. The powder XRD pattern was recorded on a Panalytical Empyrean equipped with a Cu K_{α} (λ=1.5418 Å) X-ray tube (4 0mA, 45 kV). The range of measurements was from 5° to 100° of the angle 2θ, with step size of 0.03°. ATR-FTIR spectrum was acquired on a Bruker Tensor 27 at 4 cm^{-1} resolution. The sample for STEM analysis was prepared by depositing a drop of a diluted ethanol dispersion of the $Mg(OH)_2$ powder (previously sonicated for a few seconds) on a holey carbon grid and then imaged in a Hitachi HD2700 scanning transmission electron microscope (STEM) operated at 200 kV and equipped with energy-dispersive X-ray spectroscopy (EDX) for elemental analysis.

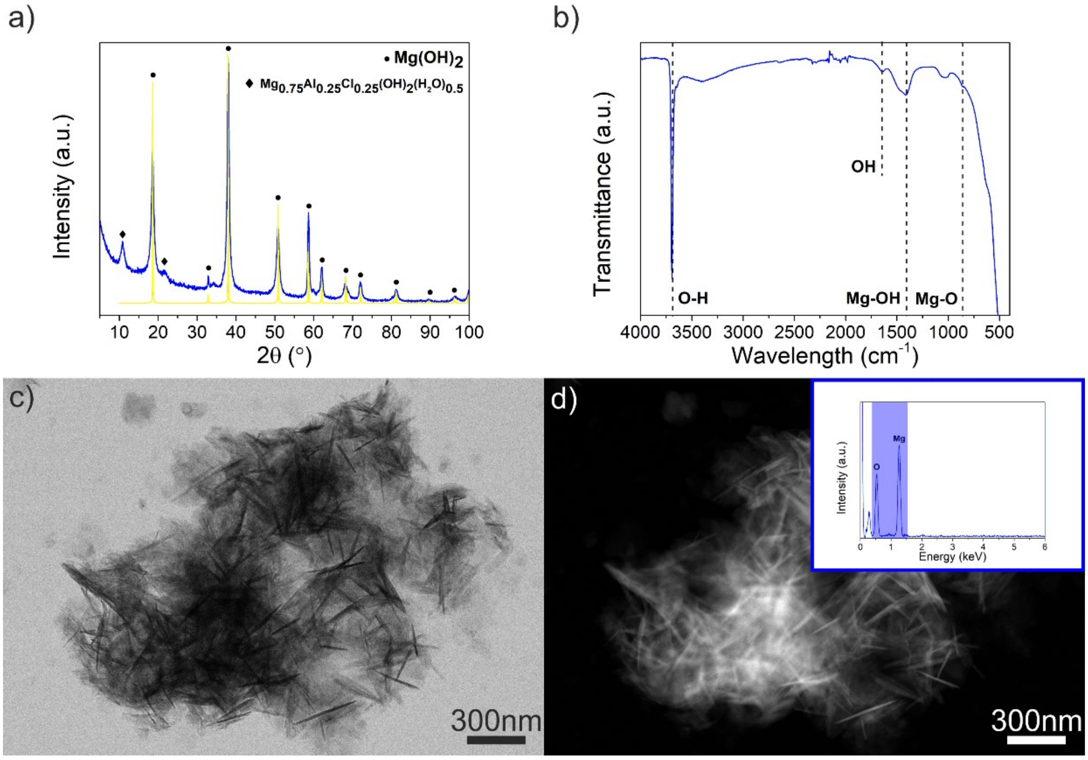

Figure S12. (a) Powder XRD pattern and (c) ATR-FTIR spectrum of the degradation products. The peaks marked by black dots were indexed to the hexagonal structure for magnesium hydroxide ($Mg(OH)_2$) according to JCPDS Card 04-016-4785 (yellow pattern). The two small peaks marked by black rhombus index to magnesium aluminum chloride hydroxide hydrate ($Mg_{0.75}Al_{0.25}Cl_{0.25}(OH)_2(H_2O)_{0.5}$) compatible with JCPDS Card 04-024-7291. The ATR-FTIR spectrum has a sharp and intense peak at 3697.3 cm^{-1} and it is attributed to the O–H band stretch in the $Mg(OH)_2$ structure [8]. (c) TE-STEM mode image and (c) corresponding ZC-STEM mode image of the $Mg(OH)_2$ structure. The inset in (d) reveals the EDX spectrum confirming the presence of O and Mg. It can be conclude that the main degradation product is $Mg(OH)_2$.

References for Supplementary Information

[1] J. Dendooven, C. Detavernier, Wiley-VCH Verlag GmbH & Co. KGaA, 2017

[2] I. E. Oliveira, R. M. Silva, J. Rodrigues, M. R. Correia, T. Monteiro, J. Faria, R. F. Silva, C. G. Silva, *RSC Adv.*, 2022, **12**, 16419-16430

[3] E. Chason, T. M. Mayer, *Crit. Rev. Solid State and Mater. Sci.*, 1997, **22**, 1-61

[4] M. Birkholz, Thin film analysis by X-Ray scattering, Wiley-VCH Verlag GmbH & Co. KGaA, 2005

[5] A. S. Veldhuis, P. Brinks, T. M. Stawski, O. F. Göbel, J. E. ten Elshof, *J. Sol-Gel Sci. Technol.*, 2014, **71**, 118-128

[6] S. Liu, Y. Li, J. Tao, R. Tang, X. Zheng, *Crystals*, 2023, **13**, 910

[7] R. Hussin1, K.L. Choy, X. Hou, *Adv. Mater. Res.*, 2016, **1133**, 352-356

[8] L. I. Fockaert,T. Würger,R. Unbehau, B. Boelen, R. H. Meißner, S. V. Lamaka, M. L. Zheludkevich, H. Terryn, J.M.C. Mol, *Electrochim. Acta*, 2020, **345**, 136166
